# Hormonal Treatment of Men with Nonobstructive Azoospermia: What Does the Evidence Suggest?

**DOI:** 10.3390/jcm10030387

**Published:** 2021-01-20

**Authors:** Ettore Caroppo, Giovanni M. Colpi

**Affiliations:** 1 Asl Bari, PTA “F Jaia”, Andrology Outpatients Clinic, 70014 Conversano (BA), Italy; 2 Andrology Unit, ProCrea Institute, 6900 Lugano, Switzerland; gmcolpi@yahoo.com

**Keywords:** nonobstructive azoospermia, micro-TESE, FSH treatment, hormonal treatment, testosterone level

## Abstract

Hormonal stimulation of spermatogenesis prior to surgery has been tested by some authors to maximize the sperm retrieval yield in patients with nonobstructive azoospermia. Although the rationale of such an approach is theoretically sound, studies have provided conflicting results, and there are unmet questions that need to be addressed. In the present narrative review, we reviewed the current knowledge about the hormonal control of spermatogenesis, the relationship between presurgical serum hormones levels and sperm retrieval rates, and the results of studies investigating the effect of hormonal treatments prior to microdissection testicular sperm extraction. We pooled the available data about sperm retrieval rate in patients with low vs. normal testosterone levels, and found that patients with normal testosterone levels had a significantly higher chance of successful sperm retrieval compared to those with subnormal T levels (OR 1.63, 95% CI 1.08–2.45, *p* = 0.02). These data suggest that hormonal treatment may be justified in patients with hypogonadism; on the other hand, the available evidence is insufficient to recommend hormonal therapy as standard clinical practice to improve the sperm retrieval rate in patients with nonobstructive azoospermia.

## 1. Introduction

Azoospermia, defined as the absence of sperm in the ejaculate, affects about 10–15% of infertile men, and in about two-third of cases is due to severe spermatogenic dysfunction [1]: such a clinical condition is termed nonobstructive azoospermia (NOA) to differentiate it from the less severe (in terms of spermatogenesis impairment) form of azoospermia due to obstruction of the seminal tract. Men with NOA may still have residual focal areas of spermatogenesis that could enable them to father children genetically of their own if mature sperm are surgically retrieved and used for intracytoplasmic sperm injection (ICSI): however, sperm retrieval is successful in up to 58% of cases, even when the most effective surgical technique, namely, microdissection testicular sperm extraction (micro-TESE), is used [2]. Among the strategies sought to maximize the sperm retrieval yield, hormonal stimulation of spermatogenesis prior to surgery has been tested by several authors. Although the rationale of such an approach is theoretically sound, studies in the field have provided conflicting results, so that the beneficial effect of hormonal optimization of spermatogenesis is yet to be demonstrated. The present narrative review is intended to discuss the evidence in the field and to offer some points for reflection for further studies. To provide unbiased results and avoid the possible impact of less effective surgical procedure on the sperm retrieval rates, only studies evaluating patients undergoing the gold-standard surgical technique for sperm retrieval (micro-TESE) [2] have been included in the present review.

## 2. Hormonal Control of Spermatogenesis

The role of follicle-stimulating hormone (FSH) in the modulation of spermatogenesis has been a matter of debate since a study on five men with inactivating mutation of the FSH receptor (FSHR) gene showed that none was azoospermic and that two had children [3]. This finding prompted some researchers to hypothesize that FSH was not necessary for spermatogenesis, but the finding that men with inactivating mutations in FSH beta subunit were completely azoospermic [4] challenged that hypothesis. Further studies clarified that the mutant FSHR is not completely inactive [5], so that a residual FSH action could be able to promote spermatogenesis and that mutations in the FSH gene are more severe than those of the FSHR [6].

Studies in mice lacking FSH (FSHKO) or FSHR (FSHRKO) clearly demonstrated that FSH is required to increase the number of spermatogonia and spermatocytes [7] and that FSH treatment was found to increase spermatogonial and spermatocyte number in hypophysectomized or gonadotropin-releasing hormone (GnRH)-immunized adult rats [8]. FSH acts also as a survival factor for spermatogonia, since acute FSH suppression induces spermatogonial apoptosis [9] and is required to stimulate the prenatal and prepubertal proliferation of Sertoli cell, an effect which is totally independent from luteinizing hormone (LH) action, as demonstrated in hypogonadal LH receptor null mice [10], as well as from testosterone action, as demonstrated in mice lacking Sertoli cell androgen receptor (SCARKO) and FSHR, which had a Sertoli cell number comparable to that of FSHRKO mice [7]. In the absence of FSH or FSHR, the Sertoli cell number is decreased by about 30–45% in comparison to normal testicular development: since the Sertoli cell is able to support a certain number of germ cells, the number of Sertoli cells determines the quantity of sperm produced. This may explain why FSHRKO mice present with complete spermatogenesis, but the amount of germ cells is lower than in wild-type animals [7].

Studies in men with congenital hypogonadotropic hypogonadism suggest that pretreatment with FSH alone prior to combined gonadotropin treatment enhances spermatogenesis [11]. However, FSH alone is not able to promote spermatogenesis beyond the pachytene spermatocytes: a recent study on SCARKO mice demonstrated that Sertoli cell androgen receptor (AR) signaling is required for the survival of meiotic prophase spermatocytes, since SCARKO mice exhibited loss of meiotic germ cells and failure of surviving spermatocytes to progress. Early meiotic prophase events are not dependent upon androgen signaling, therefore, chromosome synapsis and recombination occurred normally in surviving spermatocytes that entered meiotic prophase; however, SCARKO pachytene spermatocytes were found to acquire aberrant transcriptomic attributes (leptotene or zygotene transcriptome state) and failed to progress to subsequent transcriptomic signatures [12].

FSH alone has been also found to maintain spermatogenesis independently from testosterone; this is the case of transgenic male mice with activating FSHR mutation that enabled strong FSH activation (cAMP response > 10-fold above basal). Use of the antiandrogen flutamide to interfere the binding of androgens to the AR had no effect on spermatogenesis [13].

In normal conditions, however, testosterone signaling is required for spermatogenesis to proceed beyond meiosis. Testosterone signaling contributes also to maintaining tight junctions between adjacent Sertoli cells (essential for the blood-testis barrier) and a specialized environment for germ cells, mainly through its modulation of micro-RNAs that target genes essential for cell junction restructuring and Sertoli-germ cell adhesion. The absence of T results in disruption of blood–testis barrier, premature detachment of developing spermatid germ cells from Sertoli cells, and block of the release of mature spermatozoa from Sertoli cells, with consequent germ cells phagocytosis by Sertoli cells [14].

Testosterone (T) is produced by Leydig cells in response to LH, and mediates its effects by the AR expressed by the Sertoli cells via classical and nonclassical pathways. In the classical (genomic) pathway, T diffuses through the plasma membrane and interacts with AR and the complex T/AR translocates to the nucleus to bind to androgen response elements (AREs) in gene promoter regions and regulates gene transcription, while in the nonclassical (nongenomic) pathway, T/AR rapidly phosphorylates the SRC kinase, resulting in the stimulation of the epithelial growth factor (EGF) receptor and the fast (within 1 min) activation of MAP-kinase cascade and the CREB transcription factor, with a resulting sustained (for at least 12 h) increased protein phosphorylation and long-term gene expression changes that are mediated by increased kinase activity [15]. Both pathways are essential for spermatogenesis: a study performed on testis explants of male Sprague Dawley rats containing intact seminiferous tubules and accompanying interstitial cells, using inhibitors to specifically block each pathway in vitro, demonstrated that both pathways are able to activate transcription of the Sertoli cell-specific Rhox5 mRNA, which is dramatically upregulated in the presence of T in vivo, and that activation of either T signaling pathway in Sertoli cells can differentially modulate germ cells gene expression [15].

It has been classically demonstrated that intratesticular testosterone (ITT) concentration are much higher (50–100-fold) than circulating levels, however, spermatogenesis may be maintained by very low ITT concentration: mice with inactivation of the LH receptor (LuRKO mice) had intact spermatogenesis despite very low ITT levels (2% of control level), but administration of the antiandrogen flutamide halted sperm maturation at the round spermatid stage [16]. In addition, a more recent study demonstrated that spermatogenesis in LuRKO mice could be normalized with exogenous testosterone that achieved a serum T concentration comparable to that of WT mice, but an ITT level less than 1.5% of the WT concentration [17]. The relationship between serum and intratesticular T levels is, therefore, far to have been clearly established, so that further studies are needed.

It has been proposed that testosterone alone could induce complete spermatogenesis without the need of FSH action; indeed, subcutaneous testosterone supplementation in male mice with hypogonadotropic hypogonadism due to Kiss1 knockout was able to restore serum and intratesticular testosterone levels, promote testicular descent, and induce complete spermatogenesis from spermatocytes to elongated spermatids, but the resultant testicular weight reached only 40% of wild-type controls, similarly to what was found in hypogonadal or GnRH KO mice treated with testosterone supplementation. [18]. Such a quantitative deficit of spermatogenesis is likely to be due to the lack of FSH.

Both FSH and testosterone are, therefore, required to promote full spermatogenesis; in addition, both hormones have synergistic effects upon spermatogenesis. FSH regulates transcripts required for normal testicular function, including StAR gene, which is essential for steroid synthesis [14], and stimulates the Sertoli cell production of androgen binding globulin, which helps maintain a high T concentration within the testes. On the other hand, testosterone is thought to modulate the oligosaccharide complexity of pituitary FSH; castration induces changes in the oligosaccharide composition of pituitary FSH both in prepubertal and adult animals, and administration of flutamide, able to interfere the binding of androgens to the AR both peripherally and at hypothalamic-pituitary level, lead to a predominance of circulating FSH glycosylation variants bearing incomplete oligosaccharides [19]. Administration of testosterone enanthate to pubertal patients does not modify the serum FSH levels, but lead to a significant increase in the proportion of FSH bearing complex oligosaccharides [20].

## 3. Relationship between Serum Hormones Levels and Sperm Retrieval

It has been clearly established that preoperative FSH levels are poorly or not predictive of successful sperm retrieval in men with NOA undergoing micro-TESE [21,22]. FSH serum levels are usually high in men with NOA, and although lower levels can be found in those patients who may theoretically benefit from hormonal treatment, such as those with testis histology revealing hypospermatogenesis (HYPO) or maturation arrest (MA) [23], they cannot be used to reliably predict these conditions before surgery [24]. Lower FSH levels are not always predictive of intact spermatogenesis in patients with NOA; on the contrary, micro-TESE has been found to be more successful in patients with higher serum FSH (>15 mIU/mL) compared to those with lower serum FSH [25], and in the subset of NOA patients with MA, normal serum FSH level was associated with the lowest chance of sperm retrieval [26]. Since basal FSH serum level is not related to spermatogenesis nor to the chance of retrieving sperm in patients with NOA, it is unlikely that it may be used to predict which patient with NOA could respond to hormonal treatment. High or very high FSH serum levels should not prevent the use of exogenous FSH to stimulate spermatogenesis; although it has been classically demonstrated that such a condition could induce desensitization of the Sertoli cell signaling [27], a more recent in vitro study using KK-1 mouse granulosa cells demonstrated that FSH receptor recycling promotes the maintenance of cell surface receptors and preserves hormonal responsiveness during exogenous FSH stimulation [28].

The situation is quite different for the predictive role of serum T on the chances of sperm retrieval, since studies in the field have reported conflicting results. We were able to individuate 14 studies [29,30,31,32,33,34,35,36,37,38,39,40,41,42] who clearly reported the relationship between serum T levels and sperm retrieval rates (SRR) (Table 1). Studies differed for study design, inclusion criteria, and patients’ characteristics. Some studies were designed to compare the SRRs of conventional TESE vs. micro-TESE, while others were sought to assess presurgical markers of sperm retrieval. In 8 out of 14 studies, the cohorts included patients with Klinefelter syndrome (KS) (1.7–36.2% of the total sample), who have been found to have better chances of successful sperm retrieval (SSR) when their presurgical T levels are normal [43,44]. In 3 studies, patients received presurgical hormonal treatment (hCG, clomiphene citrate—CC, or aromatase inhibitors—AI), while in other 3, no treatment was used; in the remainders, it is not clear whether patients received any hormonal treatment before micro-TESE.

**Figure 1:**
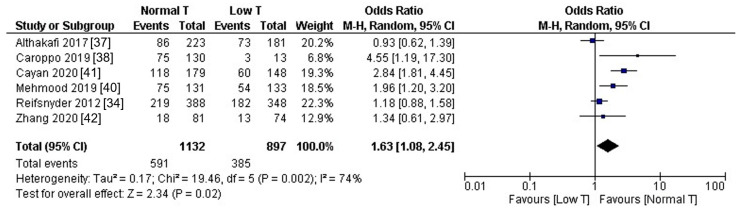
Pooled estimation of the sperm retrieval rate in patients with subnormal vs. normal testosterone level.

Seven studies included patients with subnormal presurgical T levels, and six of them [34,37,38,40,41,42] provided the sperm retrieval rates in patients with low vs. normal T levels. We pooled these latter data to compute the resulting odds ratio (OR), using random-effects models to comply with the high heterogeneity in study design, as detected by I^2^ and by Cochran’s Q. Computations and forest plot were obtained using Review Manager (RevMan, Version 5.3. Copenhagen: The Nordic Cochrane Centre, The Cochrane Collaboration, 2014). The pooled estimate, as displayed in Figure 1, showed that patients with normal T levels had a significantly higher chance of SSR compared to those with subnormal T levels (OR 1.63, 95% CI 1.08–2.45, *p* = 0.02). Notably, in the study with the largest sample size included in the analysis [34], 88% of patients with low T received hormonal treatment, but their post-treatment T levels remained still below the normal cut-off level of 300 ng/dL. However, as illustrated in the previous paragraph, the relationship between serum and intratesticular T levels needs to be fully clarified.

This is the reason why it has been suggested that ITT measurement, more than serum T level assay, could add to the evaluation of patients with NOA. Testicle aspiration is not always feasible and advisable, due to the possible inherent risks of such a procedure (pain, bleeding, infection, and testis injury), so that attempts have been made to individuate serum biomarker able to identify men with insufficient ITT and to serve for ITT levels monitoring following hormonal treatment. Since most of the circulating 17-hydroxyprogesterone (17OH-P) in men is likely of testicular and not adrenal origin, as demonstrated in orchiectomized men [45], it has been postulated that serum 17 OHP may reflect the ITT levels. Unfortunately, only a few studies tried to address this issue. Amory and coworkers evaluated 29 healthy men who received testosterone enanthate to suppress endogenous secretion before being randomly assigned to three hCG doses (125, 250, or 500 IU every other day for 3 weeks) or placebo. ITT levels were assessed by fine needle aspiration of testicular fluid; serum 17 OHP did not correlate with ITT at baseline, but following hCG treatment, a strong relationship between ITT and 17 OHP was found in men who received 250 or 500 IU hCG [46]. Very recently, Lima et al. evaluated the serum 17 OHP levels in 30 men receiving CC and/or hCG, 21 men under exogenous testosterone replacement therapy, and 42 fertile men with normal serum testosterone; despite serum T level was in the normal range in all men, serum 17-OHP was undetectable in men who received exogenous testosterone replacement therapy compared to the other two groups, and increased after CC and hCG treatment [47].

A possible role for serum insulin-like factor 3 (INSL3) as marker of ITT has been also postulated; gonadotropin suppression with exogenous testosterone and progestin resulted in decline of serum INSL3 levels compared to baseline, which was partially reversed by hCG or FSH plus hCG administration; following long-term gonadotropin suppression, serum T recovered significantly better (80% baseline) compared to serum INSL3 (38.9% baseline) [48]. In a subsequent randomized placebo-controlled clinical trial, serum INSL3 concentrations in normal men were found to dramatically decrease following acute gonadotropin suppression, and to increase in a dose–response relationship with low-dose hCG stimulation, correlating highly with ITT and serum T concentration [49].

Although intriguing, the diagnostic accuracy of 17 OHP and/or INSL3 assay as marker of ITT should be verified in larger sample studies before their introduction in the clinical practice.

## 4. Hormonal Treatment before Micro-TESE

Administration of exogenous gonadotropins has been classically found to be effective in restoring spermatogenesis in azoospermic men with hypogonadotropic hypogonadism. Consequently, hormonal treatment in men with NOA has been pursued with the aim of improving spermatogenesis before surgery, despite these patients may display high FSH and LH levels. It has been demonstrated, in fact, that in these patients Leydig cells respond to high dose hCG stimulation with increased amounts of testosterone production, even under a hypergonadotropic condition [23]. The authors demonstrated that patients with NOA display an altered gonadotrophin pulse amplitude and hypothesized that this weak endogenous gonadotrophin activity could be due to the desensitization of target cells (e.g., Sertoli and Leydig cells). Indeed, other studies demonstrated that men with NOA display abnormalities in gonadotropins pulse frequency and amplitude [50], however, these findings are presumably the consequence of an altered hypothalamus–pituitary–gonadal axis due to reduced testosterone and inhibin B feedback signaling, rather than to desensitization of target cells. As a matter of fact, it has demonstrated that desensitization of Sertoli cells does not occur, but hormonal responsiveness during FSH treatment is preserved, thanks to FSH receptor recycling [28]. Consequently, it may be hypothesized that at least a subset of men with NOA, e.g., those with subnormal T serum levels and inhibin B levels may have an altered endogenous gonadotropin secretion that justifies the use of exogenous gonadotropins or selective estrogen receptors modulators (SERMs) like CC.

Indeed, Shinjo et al. [51] found that hCG treatment significantly increased the ITT levels in patients with NOA; although ITT did not differ among those with SSR or sperm retrieval failure (SRF), men with SSR had significantly lower basal ITT levels compared to men who experienced SRF. This may reinforce the hypothesis that hormonal stimulation is required for men with subnormal T levels to optimize the sperm recovery. However, the administration of hCG alone, although being effective in improving SSR, may be not sufficient to promote spermatogenesis in men with NOA; in the same study, only men who received FSH had an increased spermatogonial proliferating cell nuclear antigen (PCNA) expression, a protein involved in nucleotide excision repair mechanisms prominently expressed in the nuclei of mitotic active spermatogonia, which has been proposed as a marker of normally active spermatogonia [52]. Furthermore, it has been demonstrated that the expression of AR on Sertoli cells increased following FSH plus hCG stimulation rather than after hCG alone [53], supporting the previous demonstration about the role of FSH in regulating Sertoli cell AR expression [54].

The results of the few studies available in this field, however, are not fully able to demonstrate a beneficial effect of hormonal treatment on the SRR in men with NOA. As displayed in Table 2, five studies [34,55,56,57,58] were carried in NOA men who underwent micro-TESE for the first time, while four [23,51,53,59] enrolled men undergoing salvage micro-TESE. The first two studies evaluated a well-selected cohort of patients, e.g., normogonadotropic men [55] and men with well-defined testis histology (MA and HYPO) [56], therefore, their results have poor generalizability, while the results of Amer and coworkers [58] are weakened by the relatively low overall SRR (32,2%), probably due to differences in skill and experience among the 15 urologists who performed micro-TESE. The two largest sample studies [34,57] provided conflicting results, i.e., in the study of Reifsnyder et al. [34], SRR did not differed among men with subnormal T levels receiving hormonal treatment (*N* = 307) or no treatment (*N* = 41), while in the study of Hussein et al. [57], SRR was significantly higher in men receiving hormonal treatment (*N* = 496) compared to those receiving no treatment (*N* = 112), and 10.9% of treated patients had sperm in the ejaculate after treatment. It has to be remarked that the post-treatment T levels differed significantly among studies, since in the study of Reifsnyder, 82% of treated patients responded to hormonal treatment with a serum T level of at least 250 ng, while in the study of Hussein, treatment was titrated to reach a target T level of 600–800 ng/dL; still, the SRR in the study of Reifsnyder in both treated and untreated patients (51 and 61%, respectively) was comparable to that obtained by patients undergoing hormonal treatment in the study of Hussein, while the SRR of untreated patients in this latter study was too low compared to the average SRR reported by studies in the micro-TESE setting.

On the other hand, the two out of four studies evaluating the results of salvage micro-TESE in treated vs. untreated patients [23,59] agreed in demonstrating the beneficial effect of hormonal treatment on SRR, however, the small number of subjects (48 treated vs. 40 untreated overall) does not allow to draw firm conclusions about that.

Another indication to hormonal treatment in men with NOA has been proposed to be their testicular histological pattern. Kato and coworkers observed that men with early MA had a lower AR index compared to those with late MA [53]; indeed, SCARKO mice have been found to display pachytene spermatocytes with aberrant transcriptomic attributes (leptotene or zygotene transcriptome state) that fail to progress to subsequent transcriptomic signatures [12]. Based on these results, Shiraishi hypothesized that only patients with late MA may respond to hormonal treatment [23]. Indeed, Aydos and coworkers did not observe improvements in SRR in patients with early MA undergoing hormonal treatment [55]. Other groups demonstrated that men with MA or HYPO respond to hormonal treatment with either the appearance of sperm in the ejaculate [57] or with improved SSR [56] even in the case of salvage micro-TESE [51]. However, also in this case, larger sample size studies are needed to confirm these findings.

The results of the available studies, although promising, are insufficient to recommend the hormonal treatment for every patient with NOA before surgery. Therefore, as stated by the recent AUA/ASRM guidelines on the diagnosis and management of infertility in men [60], patients with NOA should be informed of the limited data supporting pharmacologic prior to surgical intervention (Conditional Recommendation; Evidence Level; Grade C).

## 5. Unmet Needs and Future Directions

The management of patients with NOA is to a large extent knowledge based. Thanks to the evidence produced by the literature of the past 20 years, we know with a good approximation that about 57–60% of patients with NOA may be successful in having their testicular sperm retrieved, what clinical conditions are predictive of SSR, that SRR may vary significantly according to testis histology and that micro-TESE provides better results in terms of SRR compared to the other available surgical techniques [61]. What we do not know, due to the inconclusive data provided by the literature, is whether and how should we treat these patients before surgery to maximize the chance of sperm retrieval.

The pooled estimation of studies reporting the SRRs in patients with subnormal T compared to those with normal T levels (Figure 1) suggests that optimization of serum T levels may be indicated in hypogonadal men before micro-TESE, since it may improve the SRR. However, due to the demonstrated poor relationship between serum and intratesticular T levels [17], and to the relatively low ITT required for spermatogenesis [16], the target serum T levels to be achieved to improve spermatogenesis is not clear. Relevantly, two large sample studies [34,57] reported similar SRRs despite significantly different post-treatment T levels (230 vs. 600–800 ng/dL). To improve our knowledge in this field, it could be helpful to identify serum biomarker that could reliably predict ITT levels and serve for post-treatment ITT levels monitoring. In this perspective, the demonstration that serum 17 OHP and INSL3 levels are, to some extent, related to ITT levels, may pave the way for a new line of research. In addition, the possible predictive role of bioactive T level (computed by the formula (Bio T= free T + albumin-bound T)) on SRR would deserve further studies.

Optimization of testosterone and, possibly, ITT levels, may require also FSH, since the expression of AR on Sertoli cells increases following FSH stimulation but not with hCG [53]. In addition, since FSH is essential to promote spermatogonial proliferation in men with NOA [51], many authors added FSH to hCG or CC when falling serum FSH levels were observed following hormonal treatment (Table 2). Interestingly, although the feasibility of FSH as treatment of infertile men with oligozoospermia has been investigated by many studies, with a recent one even proposing that possible responders to FSH treatment may be identified by means of epigenetic biomarkers [62], very few studies sought to evaluate the effect of FSH alone to improve the chance of SRR in patients with NOA. Indeed, the finding that FSH may maintain spermatogenesis independently from testosterone, as found in transgenic male mice with activating FSHR mutation that enabled strong FSH activation, may prompt further studies on high dose FSH treatment of men with NOA.

Although we know with a good approximation the chance of SSR in men with different testis histology, we need more data to establish whether a specific histological pattern may be considered an indication or a contraindication to hormonal treatment. It would be helpful, therefore, for further studies in this field to report the response to hormonal treatment as stratified by testis histology. It is reminded here that, to obtain reliable testis histology pictures, the fragments of seminiferous tubules sent to the pathologist should be representative of the predominant tissue as observed at high magnification during micro-TESE.

In conclusion, to establish whether hormonal treatment may be of help in improving the reproductive potential of men with NOA, it is of the utmost importance to design studies with large sample size and well-defined entry criteria and outcome measures: in this view, collaborative multicentric studies could provide valuable data. The actual evidence is insufficient to support the indiscriminate use of hormonal treatment prior to surgery in patients with NOA.

## Figures and Tables

**Table 1:** Testosterone level and sperm retrieval in patients with nonobstructive azoospermia (NOA) undergoing microdissection testicular sperm extraction (micro-TESE).

Study	Patients Characteristics	KS	Subnormal T Levels	Main Results	Hormonal Treatment
[29]	74 patients SRR 44.4%	Yes (11/74; 14.8%)	Not shown	No relationship was found between serum T levels and SSR. Data about T serum level in patients with SSR and SRF are not provided	NS
[30]	100 patients SRR 41%	No	Not shown	T was significantly higher in men with SSR than in those with SRF (410 ± 170 vs. 320 ± 110 ng/dL. *p* = 0.0036). On multivariate logistic regression. T was predictive of SSR (OR 1.57; 95% CI 1.02–2.42; *p* = 0.042). Patients with SRF had also significantly higher FSH level and smaller testis size compared to those with SSR	NS
[31]	100 patients SRR 41%	No	Not shown	T predicted SSR at univariate logistic analysis (*p* = 0.0008) but not at multivariate logistic analysis. Data about T serum level in patients with SSR and SRF are not provided	NS
[32]	56 patients with previous failed TESA/TESE SRR 57%	Yes (1/56; 1.7%)	13 patients (23%) with T < 280 ng/dL	T was significantly higher in patients with SSR (32) compared to those with SRF (24) (458.3 ± 254.2 vs. 378.5 ± 257.3; *p* = 0.021).	NS
[33]	65 patients. SRR 56.9%	No	Not shown	T levels did not affect SSR (OR 1.06; 95% CI 0.84–1.33; *p* = 0.64). Data about T serum level in patients with SSR and SRF are not provided	NS
[34]	736 patients SRR 54.4%	Yes (88/736; 12%)	348 patients (47%) had low T levels (<300 ng/dL) before hormonal treatment. Post-treatment T levels are not provided	SRR was 52% of patients with low T vs. 56% of patients with normal T (*p* = 0.29). No difference in terms of SSR in patients with low basal T who did or did not receive hormonal treatment post-treatment T levels are not displayed.	Yes (307/348 patients with low basal T levels)
[35]	191 patients SRR 54.5%	Yes (7/191; 3%)	Not shown	Testosterone level was significantly higher in men with SSR compared to those with SRF (468 ± 263 vs. 367 ± 258; *p* = 0.023). The testosterone serum cut-off-level of 400 ng/mL significantly predicted SSR with a sensitivity of 55.2 and a specificity of 60%. AUC 0.648	NS
[36]	329 patients SRR 29.5%	Yes (65/329; 19.7%)	Not shown	T levels did not differ among men with SSR (97) and SRF (232) (420 ± 180 vs. 430 ± 190 ng/dL; *p* = 0.42)	NS
[37]	421 patients SSR 39.4%	Yes (13/431; 3%)	181 patients (43%) had low T (≤9.9 nmol/L).	SRR did not differ in patients with low T (40.3%) compared to those with normal T (38.6%) (*p* = 0.718); Mean serum T was 11.51 ± 7.40 nmol/L in patients with SSR and 11.67 ± 6.42 nmol/L in patients with SRF SRR did not differ between patients with normal T vs. untreated low T (42%. *p* = 0.526) and normal vs. pretreated low T normalized with hormonal treatment (36%; *p* = 0.736)	Yes (50/421)
[38]	143 patients SRR 55.2%	Yes (6/143; 4.1%)	13 patients (9%) had low T (300 ng/dL)	Testosterone serum level was significantly lower in patients with SRF compared to those with SSR (380 vs. 422 ng/dL; *p* = 0.007). Sperm retrieval was 23% in patients with low T and 58% in those with normal T (*p* = 0.014). However. T was not predictive of SSR in multivariate logistic analysis.	No
[39]	860 patients SSR 45.8%	Yes (312/860; 36.2%)	Not shown	Testosterone level was predictive of SSR in univariate but not in multivariate logistic regression	Yes (54/860)
[40]	264 patients 89 (33.9%) had previous surgery SRR 48.86%	NS	133 with low T (<10 nmol/L = 288 ng/dL)	SSR was 40.6 in low T vs. 57.25 in normal T (*p* = 0.0068)	No
[41]	327 patients with history of cryptorchidism SSR 52.6%	No	148 (45.2%) patients had low T (<300 ng/dL)	SSR was 40.5% in low T and 65.9% in normal T (*p* < 0.0001).	No
[42]	155 patients with idiopathic NOA SSR 20%	No	74 patients (48%) had T < 9.9 nmol/L.	SSR was 17.6 in low T and 22.2 in normal T (*p* = NS)	NS

AUC, Area under curve; KS, Klinefelter syndrome; NS, not specified; SRR, sperm retrieval rate; SSR, successful sperm retrieval; SRF, sperm retrieval failure; T, testosterone.

**Table 2:** Hormonal treatment and successful sperm retrieval (SSR) in patients with NOA undergoing micro-TESE.

Study	Patients Characteristics	Treatment	Results
[55]	108 men. 16 with SCO. 36 with focal SCO. 19 with MA. 37 with HYPO. All had serum FSH level below 8 mIU/mL	63 men received FSH 75 IU 3 times/week. 45 received no treatment	SSR 64% (40/63) in FSH treated and 33% (15/45) in controls (*p* < 0.01) SCO 2/7 (28% controls) vs. 4/9 (44% treated) *p* = NS FSCO 4/16 (25%) vs. 13/20 (65% treated) (*p* < 0.01) MA 3/8 (37%) vs. 5/11 (45% treated) *p* = NS HYPO 6/14 (42%) vs. 18/23 (78% treated) *p* < 0.05
[56]	42 men with MA (42.9%) and HYPO (57.1%)	CC 25–75 mg/day to achieve T 600–800 ng/dL (study target)	27/42 (64.3%) had sperm in the ejaculate; SSR 100% (15/15)
[34]	348 out of 736 patients had subnormal T. 307 out of 348 received hormonal therapy. 41 (12%) received no treatment	348 (47%) with low T (<300) and 388 with normal T (>300). 307 out of 348 (88%) were treated with hormonal therapy. 41 (12%) were not treated.	SSR in 52% of patients with low T and in 56% of patients with normal T. SSR 51% in treated vs. 61% in untreated
[23]	48 men with failed micro-TESE	28 hCG/hCG plus FSH if FSH levels decreased during treatment. 20 received no treatment. T did not differ among groups	Sperm retrieval 21% (treatment) vs. 0 (no treatment).
[57]	608 men	496 received CC, then hCG, and, eventually, hMG according to their response to CC, while 112 received no treatment. Target T level = 600–800 ng/dL	10.9% of patients had sperm in the ejaculate; SSR was 57% in treated and 33% in controls
[51]	20 men with failed micro-TESE	hCG followed by FSH if serum FSH < 2	SSR 3/20 (15%). T did not differ among patients with SSR and SRF Spermatogonial PCNA expression increased in patients receiving FSH Patients with SSR had significantly lower basal ITT compared to those with SRF. Post-treatment ITT increased in all patients
[53]	22 men with failed micro-TESE	All received hCG 5000 3 times a week; 12 patients received also FSH 150 thrice/week since FSH level dropped below 2	SSR 4/22 (18%). A significant increase in the AR index was observed in 12 patients receiving FSH + hCG. AR index was significantly higher in men with SSR compared to SRF. T levels did not correlate with AR index
[58]	1395 patients evaluated by different surgeons	SSR 450/1395 (32.2%) Hormonal therapy (CC or hCG or HMG or FSH or T or AI combination of drugs) in 426 patients	SSR was 27.6% (118/426) in treated vs. 31.7% (308/969) in untreated. No data about T levels in treated vs. untreated.
[59]	40 men with failed micro-TESE.	20 received testosterone for 1 month. then FSH plus testosterone, while 20 received no treatment	SSR in salvage micro-TESE was 10% in treated vs. 0 in controls. No data about T levels in treated vs. untreated.

FSCO, focal Sertoli cell only syndrome; HYPO, hypospermatogenesis; ITT, intratesticular testosterone level; MA, maturation arrest; PCNA, proliferating cell nuclear antigen; NA, not applicable; SCO, Sertoli cell only syndrome; SRF, sperm retrieval failure; SSR, successful sperm retrieval.

## Data Availability

No new data were created or analyzed in this study. Data sharing is not applicable to this article.

## List of Abbreviations

17OHP	17 hydroxyprogesterone
AI	aromatase inhibitors
AR	androgen receptor
ARE	androgen response elements
ARKO	AR knock out animals
cAMP	cyclic adenosine monophosphate
CC	clomiphene citrate
CI	confidence interval
CREB	cAMP response element-binding protein
EGF	epithelial growth factor
FSH	follicle-stimulating hormone
FSHKO	FSH knock out animals
FSHR	FSH receptor
FSHRKO	FSHR knock out animals
GNRH	gonadotropin-releasing hormone
HCG	human chorionic gonadotropin
HYPO	hypospermatogenesis
INSL3	insulin-like factor 3
ITT	intratesticular testosterone
KS	Klinefelter syndrome
LH	luteinizing hormone
LHR	LH receptor
LuRKO	LHR knock out animals
MA	maturation arrest
MAP kinase	mitogen-activated protein kinase
Micro-TESE	microdissection testicular sperm extraction
NOA	nonobstructive azoospermia
OR	odds ratio
SCARKO	Sertoli cell AR knock out animals
SCO	Sertoli-cell only syndrome
SERM	selective estrogen receptors modulators
SRF	sperm retrieval failure
SRR	sperm retrieval rate
SSR	successful sperm retrieval
STAR	steroidogenic acute regulatory protein
T	testosterone
